# Genetic polymorphisms in malaria vaccine candidate *Plasmodium falciparum* reticulocyte-binding protein homologue-5 among populations in Lagos, Nigeria

**DOI:** 10.1186/s12936-019-3096-0

**Published:** 2020-01-06

**Authors:** Olusola Ajibaye, Akinniyi A. Osuntoki, Emmanuel O. Balogun, Yetunde A. Olukosi, Bamidele A. Iwalokun, Kolapo M. Oyebola, Kenji Hikosaka, Yoh-ichi Watanabe, Godwin U. Ebiloma, Kiyoshi Kita, Alfred Amambua-Ngwa

**Affiliations:** 1 Department of Biochemistry & Nutrition, Nigerian Institute of Medical Research, Yaba, Lagos, Nigeria; 2 Department of Biochemistry, College of Medicine, University of Lagos, Idi-Araba, Lagos, Nigeria; 3 Department of Biochemistry, Ahmadu Bello University, 2222 Zaria, Nigeria; 4 Department of Biomedical Chemistry, Graduate School of Medicine, The University of Tokyo, 7-3-1 Hongo, Bunkyo-ku, Tokyo, 113-0033 Japan; 5 Parasitology and Bioinformatics Unit, Department of Zoology, Faculty of Science, University of Lagos, Akoka, Lagos, Nigeria; 6 Department of Infection and Host Defense, Graduate School of Medicine, Chiba University, 1-8-1 Inohana, Chuo-ku, Chiba, 260-8670 Japan; 7 School of Health and Life Sciences, Teesside University, Middlesbrough, TS1 3BX UK; 8 School of Tropical Medicine and Global Health, Nagasaki University, Nagasaki, 852-8523 Japan; 9 Medical Research Council at the London School of Hygiene and Tropical Medicine, Atlantic Boulevard, Fajara, The Gambia

**Keywords:** Polymorphisms, Reticulocyte-binding protein homologue-5, Haplotypes, Histocompatibility, Gene flow, Linkage, Unstructured regions

## Abstract

**Background:**

Vaccines are the most reliable alternative to elicit sterile immunity against malaria but their development has been hindered by polymorphisms and strain-specificity in previously studied antigens. New vaccine candidates are therefore urgently needed. Highly conserved *Plasmodium falciparum* reticulocyte-binding protein homologue-5 (PfRH5) has been identified as a potential candidate for anti-disease vaccine development. PfRH5 is essential for erythrocyte invasion by merozoites and crucial for parasite survival. However, there is paucity of data on the extent of genetic variations on PfRH5 in field isolates of *Plasmodium falciparum*. This study described genetic polymorphisms at the high affinity binding polypeptides (HABPs) 36718, 36727, 36728 of PfRH5 in Nigerian isolates of *P. falciparum*. This study tested the hypothesis that only specific conserved B and T cell epitopes on PfRH5 HABPs are crucial for vaccine development.

**Methods:**

One hundred and ninety-five microscopically confirmed *P. falciparum* samples collected in a prospective cross-sectional study of three different populations in Lagos, Nigeria. Genetic diversity and haplotype construct of *Pfrh5* gene were determined using bi-directional sequencing approach. Tajima’s D and the ratio of nonsynonymous vs synonymous mutations were utilized to estimate the extent of balancing and directional selection in the *pfrh5* gene.

**Results:**

Sequence analysis revealed three haplotypes of PfRH5 with negative Tajima’s D and dN/dS value of − 1.717 and 0.011 ± 0.020, respectively. A single nucleotide polymorphism, SNP (G → A) at position 608 was observed, which resulted in a change of the amino acid cysteine at position 203 to tyrosine. Haplotype and nucleotide diversities were 0.318 ± 0.016 and 0.0046 ± 0.0001 while inter-population genetic differentiation ranged from 0.007 to 0.037. Five polypeptide variants were identified, the most frequent being KTKYH with a frequency of 51.3%. One B-cell epitope, 151 major histocompatibility complex (MHC) class II T-cell epitopes, four intrinsically unstructured regions (IURs) and six MHC class I T-cell epitopes were observed in the study. Phylogenetic analysis of the sequences showed clustering and evidence of evolutionary relationship with 3D7, PAS-2 and FCB-2 RH5 sequences.

**Conclusions:**

This study has revealed low level of genetic polymorphisms in PfRH5 antigen with B- and T-cell epitopes in intrinsically unstructured regions along the PfRH5 gene in Lagos, Nigeria. A broader investigation is however required in other parts of the country to support the possible inclusion of PfRH5 in a cross-protective multi-component vaccine.

## Background

The pathology associated with *Plasmodium falciparum* malaria is elicited by erythrocyte invasion during the asexual stage of parasite development, which is an important target for vaccine development [1]. Owing to the widespread of multi-drug resistant strains of *P. falciparum* in malaria endemic regions, vaccines may be the reliable intervention strategy to mount sterile immunity against malaria through both humoral and T-cell responses.

The newly introduced malaria vaccine, the RTS, S/ASO1 (RTS,S), recently recommended by the World Health Organization (WHO) elicits some protective effect against malaria. However, its field efficacy is limited to 39% among children with uncomplicated malaria and 29% in severe malaria cases and only protective against *P. falciparum* [2]. The field efficacies of vaccine against *P. falciparum*, the deadliest species of malaria parasite, have been significantly hampered by genetic polymorphisms in candidate antigens. These polymorphisms alter the epitope expression leading to loss of vaccine efficacy [3], hence vaccines designed with sequence compositions identical to the infecting parasites can offer protection against infections by such parasites [4, 5].

Population genetic analysis of vaccine candidate antigens offers an insight into the extent and dynamics of genetic polymorphisms and guides vaccine design [4]. This often involves investigation of location and distribution of these genetic variations. However, genetically distinct *P. falciparum* strains involved in the development of strain-specific immunity are most likely carried by individuals living in malaria endemic areas [4, 6, 7].

Chemotherapy, host immunity and intensity of competitions within hosts impose selection pressure on vaccine candidate antigens [8]. Epidemiological survey of genetic diversity associated with *P. falciparum at* study sites for vaccine trials is utilized in molecular identification of prevalent genetic variants of vaccine candidate proteins [4]. *Plasmodium falciparum* reticulocyte binding-like protein family (PfRH) has been in the focus for vaccine development because it is a key determinant of erythrocyte invasion [9]. *Plasmodium falciparum* Rh comprises of five members: PfRH1, PfRH2a, PfRH2, PfRH4 and PfRH5. PfRH5 is currently a promising vaccine candidate antigens fast approaching clinical development [9]. Most of the clinical manifestations in malaria is as a result of erythrocyte invasion, which is a complex process involving merozoite attachment to red blood cell (RBC), re-orientation, tight junction formation and parasite entry into the RBC. The receptor PfRH5 on the RBC is Basigin (CD 147).

PfRH5 is a 63-kD protein, encoded by the *rh5* gene located on chromosome 4. The protein is located at the tight junction together with its associated binding proteins, AMA1 and RON2. PfRH5 lack trans-membrane domain in its sequence, its binding with AMA1 and RON2 may be important for its membrane anchorage. Antibodies against PfRH5 from different parasite strains, inhibited the growth of different strains of *P. falciparum*, out-performing other antigens on the vaccine development platforms [9].

Nonetheless, limited genetic polymorphisms have been reported on *pfrh5* gene, however, these SNPs significantly modify the protein affecting its binding to basigin and in the active complex. Certain peptides in PfRH5 have been identified as having high red blood cell binding affinity (HABPs) [10]. They include the HABPs 36718, 36727 and 36728 in which genetic mutations affect the protein structure of the whole PfRH5 protein [10]. Currently, a total of five informative non-synonymous SNPs have been reported in *Pf*RH5 [10] but there is a paucity of information on PfRH5 polymorphisms among *P. falciparum* isolates circulating in most African countries.

This cross-sectional study aimed to evaluate the status and extent of polymorphisms in PfRH5 HABPs 36718, 36727 and 36728 among *P. falciparum* isolates from Lagos, Nigeria. The study also investigated the presence of B- and T-cell epitopes that are immunologically important with respect to effective malaria vaccine design.

## Methods

### Sampling and study design

**Fig. 1:**
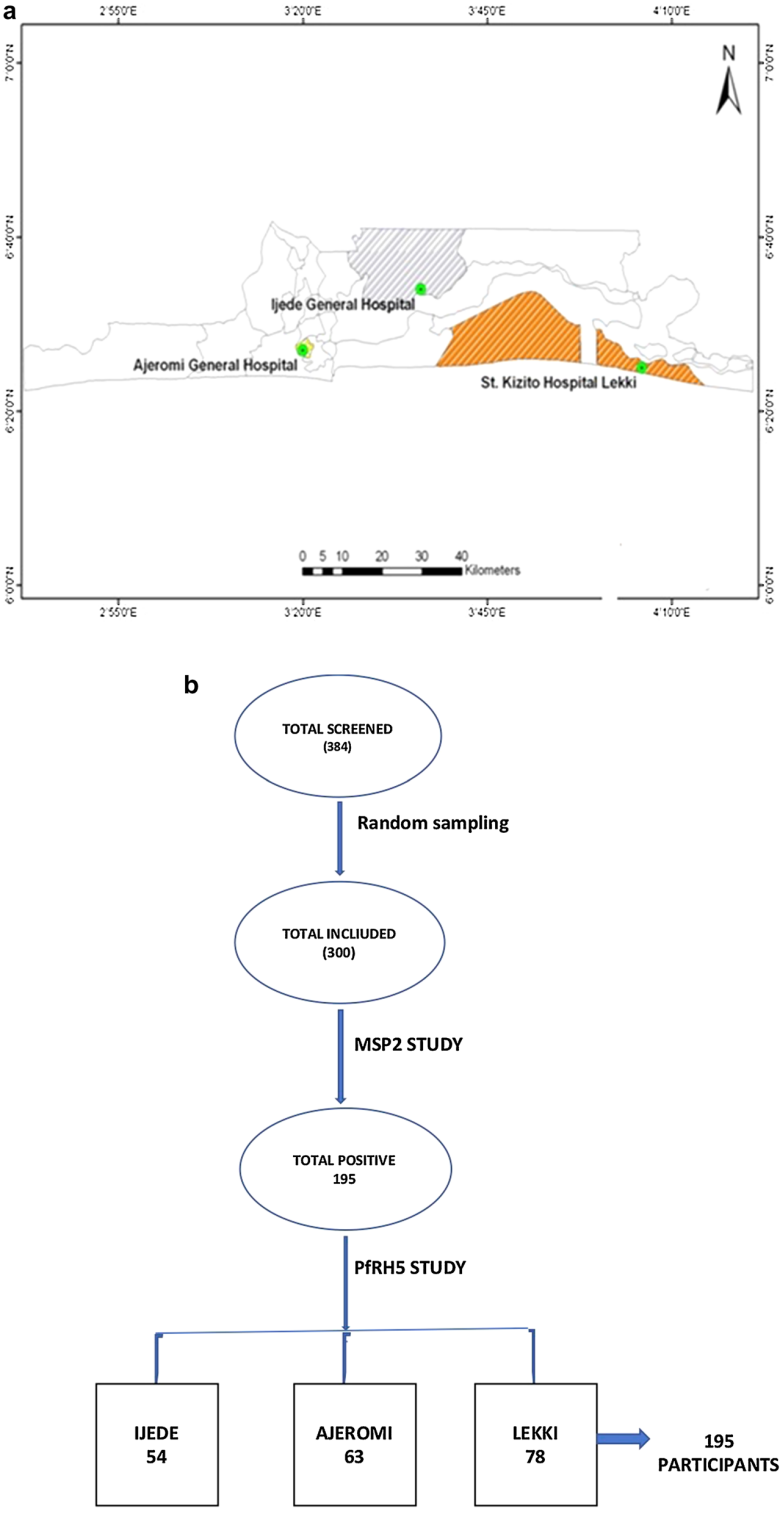
**a** Geographical location of the three study sites. **b** Participant sampling

In this cross-sectional study, participants were recruited at the peak of malaria transmission from March to September 2016 at three health facilities in Lagos State, South-West Nigeria. The study areas included (a) Ijede General Hospital, Ijede, (b) Ajeromi General Hospital, Ajegunle, (c) St. Kizito’s Primary Healthcare Centre, Lekki (Fig. 1a). Malaria transmission is high in these areas and occurs all-year round, the selected facilities have over 500 outpatients per week. Informed consent (parental consent and/or assent from under-16 participants) were obtained from the patients presenting at these centres with symptoms suggestive of malaria (axillary temperature ≥ 37.5 or history of fever in the last 48 h). Participants with *P. falciparum* mono-infection (infection by *P. falciparum* alone as confirmed by microscopy and pan-specific SD Bioline malaria rapid diagnostic test kit, Wagle, India) and a minimum of 2000 *P. falciparum* parasites/µL estimated by thick film examination were enrolled into the study, after which they were treated with artemether-lumefantrine according to national guidelines [11]. Finger-pricked blood samples collected from the participants (Fig. 1b) were used to prepare smears according to WHO standard protocol [12].

The slides were independently read by two microscopists for the estimation of parasite densities and species determination using the thick and thin blood films, respectively, assuming an average white blood cell count of 8000/μL. gDNA extraction, parasite genotyping and polymerase chain reaction (PCR) Amplification of *pfrh*5 gene were done at the Malaria Research and Immunology Laboratory of the Nigerian Institute of Medical Research, Lagos, Nigeria. DNA re-amplification, cleaning and sequencing of *pfrh*5 were done at the Department of Biomedical Chemistry, Graduate school of Medicine, University of Tokyo, Tokyo, Japan.

### DNA extraction and parasite clonal differentiation

Extraction of whole-blood genomic DNA (gDNA) was carried out using QIAGEN QIAamp^®^ DNA Extraction mini-kit (Qiagen, UK, CAT NO: 51306) from the individual dried blood spots. The extracted DNA samples were stored at − 20 °C until PCR. The quality as well as quantity of each DNA sample was determined using Nanodrop *1000* (Thermo Fisher Scientific, USA). To differentiate the parasites based on clonal origin, oligonucleotide primers (MSP2-OF:5′-ATGAAGGTAATTAAAACATTGTCTATTATA-3′ and MSP2-OR: 5′-CTTTGTTACC-ATCGGTACATTCTT-3′ for the primary reaction. The secondary reactions involved: FC27-OF:5′-AATACTAAGAGTGTAGGTGCARATGCTCCA-3′ and FC27-OR: 5′-TTTTATTTGGT GCATTGCCAGAACTTGAAC-3′, IC/3D7M2-F: 5′ AGAAGTATGG-CAGAAAGTAAK CCTYCTACT-3′and IC/3D7M2-R:5′-GATTGTAATTCGGGGGATTTGTTCG-3′ from published sequences in the GenBank [13], were used to amplify the block 3 of MSP-2 in nested-PCR. The conditions for the primary reactions were an initial denaturation step at 95 °C for 5 min, followed by 30 cycles consisting of 1 min of denaturation step at 94 °C, 2 min of annealing at 55 °C, and 2 min of extension at 72 °C, followed by a final extension at 72 °C for 5 min. The same cycling conditions were used for the secondary PCR (Nest 2) reaction except that the extension in the cycle was for 1 min. The DNA regions were amplified in 50 µL reaction mixtures containing: 1.25 U PfuUltra II Fusion HS DNA polymerase (Agilent Technologies, Santa Clara, USA), 1× polymerase reaction buffer, 2.5 mM MgCl_2_, 0.25 mM dNTPs and 1 µM of each primer. Each amplification with conserved or family-specific primer pair being done separately as previously described [14], using 2 μL of 5 ng/μL DNA sample. PCR products were electrophoresed on 1.5% agarose gels, and DNA bands visualized with the gel documentation system (Infinity 3026, Vilber Lourmat, Marne-la-Vallée, France) after ethidium bromide staining. The sizes of the fragments obtained were estimated by comparison to the 1.3 Kb (100 bp) DNA ladder (Jena Bioscience GmbH, Germany) as marker in comparison with standard purified genomic DNA from 3D7 laboratory strains similarly electrophoresed.

### Amplification of *pfrh5* gene

*Plasmodium falciparum* reticulocyte binding protein homologue 5 (*pfrh5*) gene was amplified from the whole-blood genomic DNA of samples with single parasite line using the primer set sequences namely: RH5-F1: 5′-ACCCATGAGGAATTGAGTC-3′ and RH5-R1: 5′-CGGTTTCATCATCTGTCTC-3′ amplifying the exon region encoding HABPs 36718, 36727 and 36728 [10]. The cycling parameters for the PCR were: 10 min initial denaturation at 94 °C followed by 35 cycles of 1 min denaturation at 94 °C, 1 min annealing at 55 °C, 2 min extension at 72 °C and a 10 min final extension at 72 °C. All PCR reactions were carried out with 2 μL of DNA in a total volume of 50 μL containing 0.2 mM dNTPs, 2 mM MgCl_2_, 1 μM of each primer, and 1 unit of PfuUltra II Fusion HS DNA polymerase (Agilent Technologies, Santa Clara, USA). The gel slices containing the DNA fragment of interest were excised and purified with the MagExtractor- PCR & Gel Clean up kit (TOYOBO Co. LTD., Osaka, Japan).

The purified DNA was sequenced using both forward and reverse primers after precipitation using an ABI3777 automatic DNA sequencer (Applied Biosystems). After sequencing, the gene sequences were edited using the Bioedit software and aligned amongst themselves using CLUSTAL W in MEGA 6 [15]. Blast searches were done to compare the RH5 haplotypes with the corresponding sequences available in the Genbank database and also with *P. falciparum* 3D7 reference RH5 sequence (GenBank accession number XM_001351508.1). The sequences were deposited in the GenBank database of NCBI (National Centre for Biotechnological Information) (GenBank: KX418644–KX418646).

### Genetic analyses

The number of segregating sites (S), observed nucleotide diversity per site between any two sequences assuming that the samples are random was estimated as (∏), number of haplotype (H: uninterrupted sequence of nucleotides or amino acid sequence variants using the PfRH5 locus of the *P. falciparum* 3D7 strain (GenBank accession number: XM_001351508.1) as the reference sequence for all the comparisons), number of mutations, haplotype diversity (Hd), Tajima D (positive values show excess of alleles and balancing selection), the ratio of the rate of non-synonymous to synonymous mutations (dN/dS) (≪1 values are indication of negative selection), Linkage disequilibrium, haplotype prevalence, Estimate of recombination effect ‘R’ between adjacent sites (to assess the rate of recombination which may influence Tajima’s D) were determined using DnaSP version 5.10.1 while phylogenetic analysis was done by neighbour-joining (NJ) method with Kimura-2-parameter distance matrix in MEGA 6.

### Immunological epitopes (B- and T-cell)

The ABC predserver [16] using a threshold of 0.81 and the Human Leukocyte antigens (HLA) binding prediction algorithm (Tmhc Prediction server) were used to predict the PfRH5 linear B-cell epitopes and screen the PfRH5 sequences for potential CD4 +/CD8 + epitopes with respect to related HLA. Major histocompatibility complexes (MHC-I and MHC-II) display specificity for binding with their respective epitopes. In humans, these MHC molecules are known by HLA alleles. The HLA binding peptides were predicted (threshold setting = 3.000) using the Tmhc Pred server. This prediction is based on the virtual and quantitative matrices based on 97 MHC alleles using position specific scoring matrices (PSSMs) and utilizes supervised learning method called support vector machine [16]. The relative binding ability of the different peptides to specific MHC molecule were assessed. The peptide concentrations that leads to 50% inhibition, IC50, were taken. The top binders with the cut-off value of IC50 < 100 nM were shortlisted and those with IC50 < 50 nM selected to check the potential impact of the SNPs with respect to alteration of the core sequences of lead epitopes.

The intrinsically unstructured regions (IURs) were screened using the Biocomputing package for Accurate Detection of Protein Disorder (Cspritz) server [17].

## Results

### Demographic profile of patients

Three hundred and eighty-four febrile subjects comprising 185 males and 199 females were positive for *P. falciparum* malaria out of the 1883 examined in the three study areas. Of the 384 microscopically confirmed malaria patients, 41.93% were < 5 years, 300 (100 per site) were included in this study. Overall, median age was 10 years and prevalence of *P. falciparum* malaria among the febrile participants was 20.39% ordered as Ijede > Ajeromi > Lekki (Table 1). Approximately 36.25% of the uninfected healthy controls included were < 5 years (Table 1). Three hundred individuals (≥ 1 year old) were randomly selected for genetic analysis.

**Table 1:** Demographic profile of study population

Profile	Ijede	Lekki	Ajeromi	Total
Number of patients tested for *P. falciparum* malaria	480	616	787	1883
Number of patients microscopically positive for malaria	114	117	153	384
Prevalence of symptomatic malaria (%)	23.75	18.99	19.44	20.39
Sex of participants				
Test				
Male	61	54	70	185
Female	63	55	81	199
Control				
Male	11	14	15	40
Female	11	14	15	40
Participants age groups				
Test (years)				
< 5	32	51	78	161
≥ 5	82	66	75	223
Control (years)				
< 5	8	10	11	29
≥ 5	14	18	19	51
Parasite density (parasite/μL) (years)				
< 5	145,629	128,302	134,870	136,267 ± 8748.23^a^
≥ 5	45,595	20,040	33,101	32,912 ± 12,778.54^a^

Overall, prevalence of malaria among the febrile participants was 20.39%, in the ordered Ijede > Ajeromi > Lekki

^a^Mean value ± SD

### Parasite clonal distribution

**Fig. 2:**
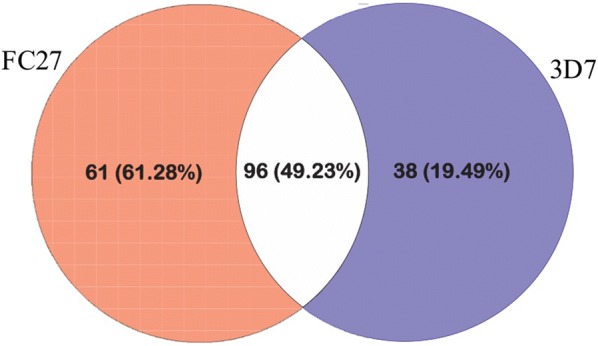
Distribution of MSP2 alleles in isolates from the sites studied

Of the 300 isolates included in this study, 195 were PCR positive for MSP2 *(*showing bands for one or both families). Quality assessment of the extracted DNA showed an average yield of 80 ng/µL and purity 1.86 (OD 260/280). Nested PCR analysis of the MSP-2-confirmed samples revealed that both 3D7 and FC27 families of *P. falciparum* MSP-2 were present in the study sites (Fig. 2).

### Polymorphisms and haplotypic variations of PfRH5

**Fig. 3:**
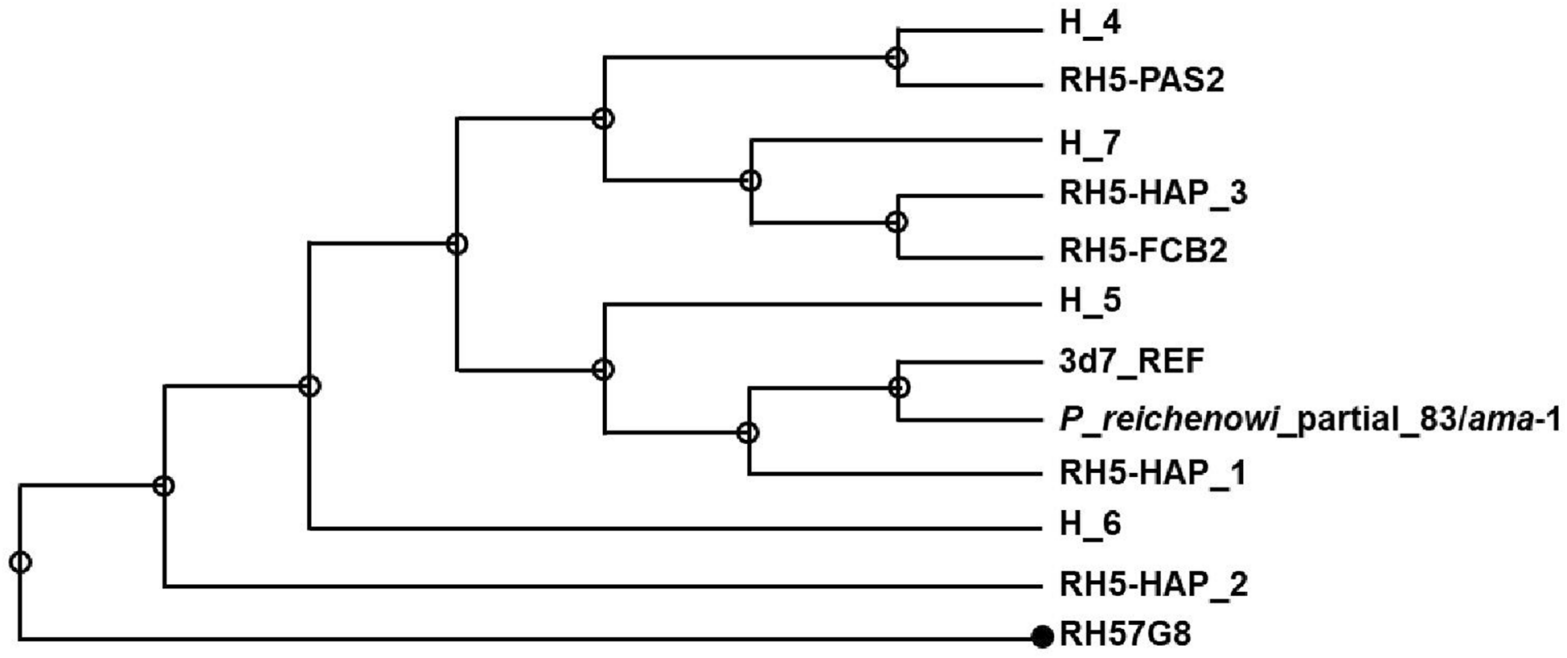
Molecular phylogenetic analysis of RH5 haplotypes by Maximum Likelihood method. A neighbour-joining (NJ) tree depicting the relationship between different RH5 haplotypes observed among Nigerian *P. falciparum* populations and with other RH5 sequences including PAS-2, FCB-2, 7G8 and 3D reference strain RH5. The evolutionary history was inferred by using the Maximum Likelihood method. The analysis involved 78 nucleotide sequences. Numbers below the lines indicate percentage bootstrap values for 1000 replications. All positions containing gaps and missing data were eliminated. Evolutionary analyses were conducted in MEGA6 [15]. The RH5 sequence of *Plasmodium reichenowi* was used as an out-group

Analysis of the HABPs 36718, 36727 and 36728 of PfRH5 revealed 810 bp sequence (encoding amino acid residues 23–241 of PFRH5) of which 795 sites were invariable or monomorphic and 15 (1.85%) were polymorphic (6 singleton and 9 parsimony informative) sites. A total of 5 non-synonymous SNPs were found in the populations studied described as: K62R, T81Q, P197S, C203Y and H240R with the mutation C203Y being the most prevalent with 74.87%. Of these resultant 5 amino acid changes, 7 peculiar polypeptide variants were observed, and defined based on the amino acid composition at the 5 variable residue positions with the most frequent variant (variant 1: KTKYH) having a frequency of 51.3% and the least (variant 7: ENKCR) with a frequency of 0.5% (Table 2). Three different RH5 haplotypes (H1, H2 and H3) were found among the *P. falciparum* populations studied based on these variants (Table 4). A single nucleotide change (G608A) was observed in the major polymorphic nucleotide of PfRH5, HABP 36727, (according to the 3D7 reference strain numbering) corresponding to amino acid change at residue 203 from Cysteine to Tyrosine (Additional file 1: Table S1). The haplotype diversity (Hd), nucleotide diversity (π) and average number of pairwise nucleotide differences within population showed no clear evidence of regional variation (Table 3). The sequences reported in this study have been deposited in the database (GenBank: KX418644–KX418646). Phylogenetic analysis showed evolutionary relationship of the haplotypes with reported PfRH5 around the world (Fig. 3) using *Plasmodium reichenowi* as the out-group.

**Table 2:** Frequency of 7 PfRH5 variants of 5 SNPs in Nigerian isolates

Polypeptide	Variant	K62R	T81Q	K197N	C203Y	H240R	Frequency (%)
KTPYH	1	K	T	K	Y	H	51.3
RQNCR	2	R	Q	N	C		24.0
KTPYR	3	K			Y		20.2
KTPYH	4				Y		2
KTPCH	5				C		1
KTPNR	6				N		1
ENPCR	7					R	0.5

**Table 3:** Measures of DNA sequence polymorphisms at the HABP 36727 of RH5 among Nigerian *P. falciparum* populations (N = 195)

Study areas (n = 195)	Segregating sites (S)	Singleton variable sites	Parsimony informative sites	Total no of mutation	K	H	Hd ± S.D	Π ± S.D	dN/dS ± S.E	Tajima’s D
Ijede (n = 54)	13	13	0	13	5.20	3	0.403 ± 0.024	0.0064 ± 0.0058	0.017 ± 0.005	− 1.210
Lekki (n = 63)	11	11	0	11	7.53	2	0.671 ± 0.314	0.0091 ± 0.0012	0.013 ± 0.313	− 0.837
Ajeromi (n = 78)	9	8	1	10	7.33	2	0.509 ± 0.027	0.0080 ± 0.0070	0.009 ± 0.015	− 0.843
Total (n = 195)	15	6	9	15	3.727	3	0.318 ± 0.016	0.0046 ± 0.0001	0.011 ± 0.020	− 1.717

*S* number of segregating (polymorphic/variable) sites, *K* average number of pairwise nucleotide differences, H number of haplotypes, *Hd* Haplotype diversity, *π* observed average pairwise nucleotide diversity, *dN/dS* rate of non-synonymous mutations minus rate of synonymous mutations, *D* Tajima’s D test statistics

S.E was used in place of SD to correct for extraneous values of dN/dS

### *Plasmodium falciparum* genetic structure, evidence of selection and recombination

**Fig. 4:**
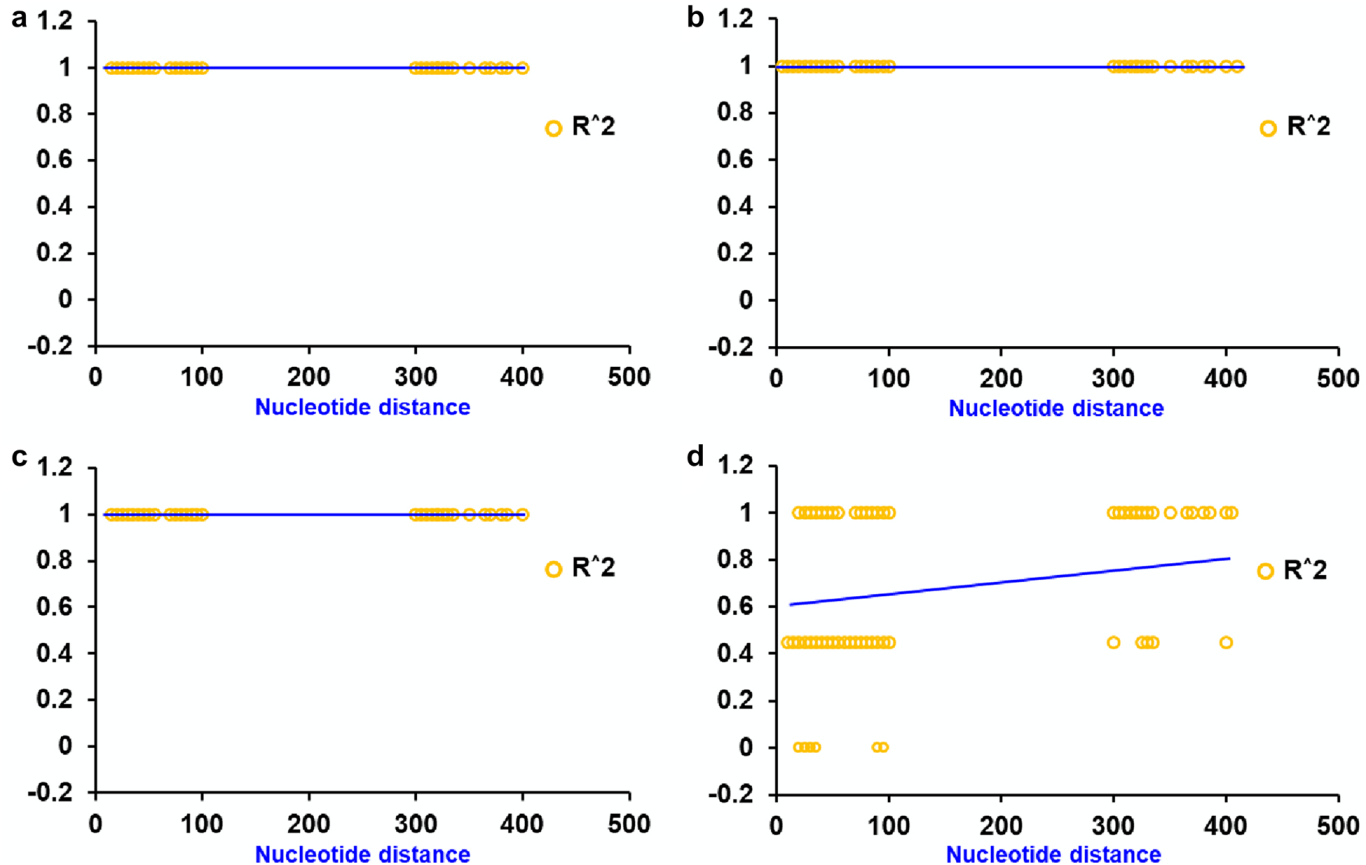
Linkage disequilibrium (LD) plot showing non-random association between nucleotide variants at different polymorphic sites on RH5. The R^2^ values are plotted against the nucleotide distances with two-tailed Fisher’s exact test of significance using DnaSP. The value of LD index (range from − 1 to +1) increased with increasing distance, indicating no recombination events taking place. All polymorphic sites were considered in the analysis. **a** Ajeromi, **b** Lekki, **c** Ijede, **d** total

The LD (Fig. 4) and dN/dS values for PfRH5 showed negative selection with negative values for Tajima’s D and values < 1 for dN/dS (Table 3). Based on the three PfRH5 haplotypes (H1, H2 and H3), inter-population nucleotide differences (Kxy) varied from 10.662 (between Ijede and Ajeromi) to 11.939 (between Lekki and Ajeromi). Similarly, the average number of nucleotide (Dxy) and net nucleotide substitution per site (Da) between populations ranged from 0.0233 to 0.0261 and 0.00015 to 0.00096, respectively (Table 4).

**Table 4:** Inter-population genetic differentiation of *P. falciparum*

S/N	Population 1	Population 2	Kxy^a^	Dxy^b^	Da^c^	Fst^d^
1	IJEDE	LEKKI	11.535	0.0252	0.00089	0.035
2	IJEDE	AJEROMI	10.662	0.0233	0.00015	0.007
3	LEKKI	AJEROMI	11.939	0.0261	0.00096	0.037

^a^Average number of nucleotide differences between populations

^b^The average number of nucleotide substitutions per site between populations

^c^The net nucleotide substitutions per site between populations

^d^Fixation index, a measure of genetic differentiation between population (range from 0 to + 1)

### Variability of Immunological (B- and T-cell) epitopes in PfRH5

**Fig. 5:**
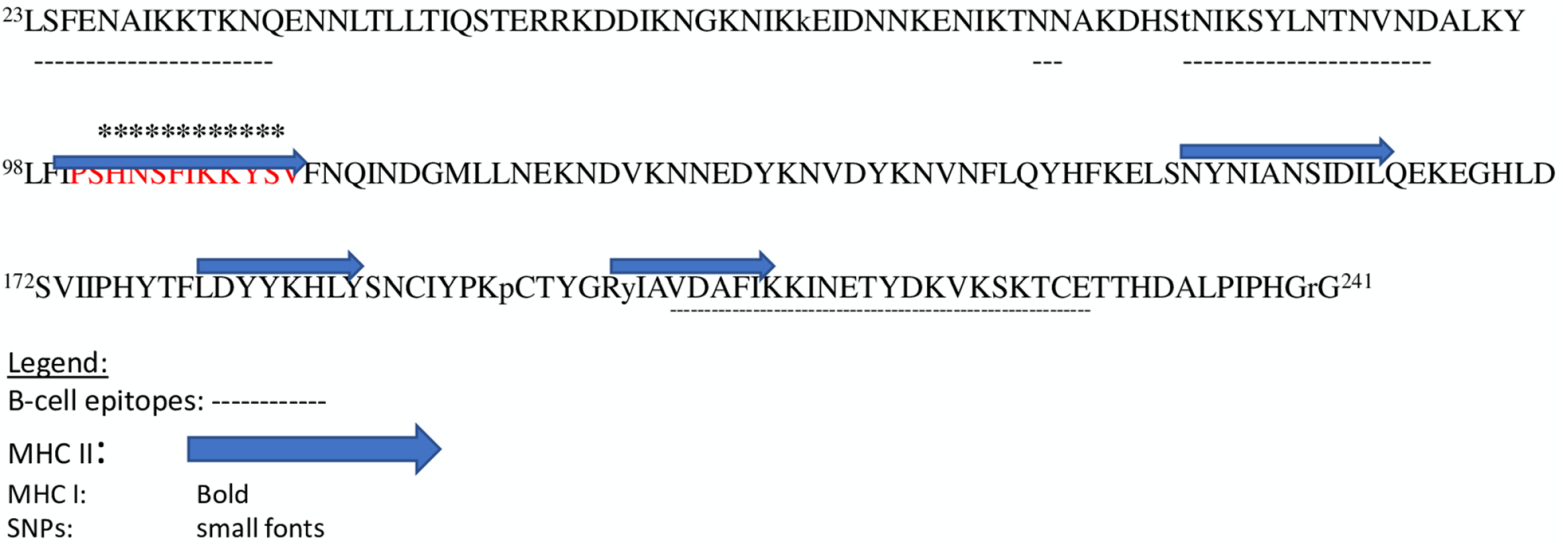
Distribution of SNPs, B- and T-cell epitopes on PfRH5 from Nigerian *P. falciparum*

To investigate the representativeness of specific predicted epitopes on the basis of their immunological significance, in comparison with the complete active PfRH5 protein, B- and T-cell epitopes and the IURs were screened for genetic mutations. This analysed the complexity of genetic diversity of predicted epitopes. One B-cell epitope, 151 MHC class II T-cell epitopes, four IURs (highly disordered) and six MHC class I T-cell epitopes were observed (Fig. 5, Table 5). Two out of the 5 genetic mutations in the *pfrh5* genes were found located in the epitope region, however, there was no commitment to populating genetic mutations in epitope regions along the gene sequences mapping the *pfrh5* HABPs (x^2^ = 6.72, P = 0.062, df = 2). A large part of the gene sequences was conserved, of which some of the epitopes were located in the disordered region of the protein (Fig. 5).

**Table 5:** Predicted CD8 + T-cell, CD4 + T-cell, and B-cell epitopes in PfRH5 (IC50 < 50 nM)

Allele	Predicted peptide	IC50	Number of polymorphic sites
T-cell epitopes			
Class II			
DRB1_1304	LNTNVNDAL	8.72	0
	TNVNDALKY	30.21	0
DRB1_0701	YLFIPSHNS	12.81	0
	FKELSNYNI	27.83	0
	YNIANSIDI	46.31	0
	YKHLYSNCI	47.83	0
	YIAVDAFIK	48.32	1
DRB1_1101	LTLLTIQST	21.37	0
	IKSYLNTNV	41.22	0
	FIKKYSVFN	44.67	0
	FNQINDGML	48.35	0
	FKELSNYNI	48.56	0
	YGKYIAVDA	48.64	1
DRB1_0120	FNQINDGML	31.84	0
	YNIANSIDI	37.21	0
	LKYLFIPSH	45.57	0
	YKHLYSNCI	47.87	0
Class I			
HLA-A*30:01	KTKNQENNL	14.79	0
	IAVDAFIKK	15.87	0
	KVKSKTNET	36.71	0
	SVIIPHYTF	29.00	0
HLA-B*35:01	PHYTFLDY	15.36	0
	FIKKINETY	40.42	0
B-cell epitope	PSHNSFIKKYSV	0	

## Discussion

In natural *P. falciparum* populations, one major hindrance to effective vaccine design and development is the sequence variation on the surface of vaccine candidate proteins. Therefore, a vaccine targeting a single allelic form of parasite proteins cannot elicit required protective immunity [18].

The difficulty of developing an effective malaria vaccine is largely due to polymorphisms in candidate proteins resulting strain-specificity and failed field trials. However, *Pf*RH5 is highly susceptible to cross-strain neutralizing vaccine-induced antibodies, and out-performs all other antigens delivered by vaccine platforms [19, 20]. Moreover, only limited polymorphisms have been reported in PfRH5. Hence, there is need for population-specific investigation of sequence diversity of PfRH5 in Nigeria to inform design of effective vaccine.

This study investigated the extent of polymorphisms in *P. falciparum* RH5 in Lagos, Nigeria to improve understanding of the population-specific processes of acquired immunity and vaccine failure.

HABPs 36718, 36727 and 36728 of PfRH5 were genetically analysed from Nigerian isolates. Among the genetic mutations and amino acid changes found, five non-synonymous SNPs were observed giving rise to 5 polypeptide variants with frequencies ranging from 51.3 to 84.9%.

Genetic analysis of the 810 bp PfRH5 sequences revealed that Tajima’s D and dN/dS values for PfRH5 were negative suggesting a purifying selection on this gene. Consequent upon the very low polymorphisms in this gene, there is a high tendency for susceptibility of Nigerian isolates of *P. falciparum* to universally effective vaccines based on PfRH5 [21] reported a rationale to advance *Pf*RH5-based vaccine to the Phase I/IIa human trials, which if successful, could be combined with vaccines targeting other stages of the *P. falciparum* life cycle, such as the sporozoite stage in the RTS, S vaccine. This promises a good efficacy of such vaccines going by the results from this study.

Phylogenetic analysis revealed clustering of the haplotypes and evolutionary relationship of the haplotypes with the reference 3D7 and other reported strains including the PAS-2, FCB-2 RH5 sequences [21]. It has been clearly demonstrated that evolutionary analysis may help explain changes in tropism when information on the location and nature of functional genetic variation are provided [22, 23].

The low *Fst* values suggest high gene flow between two populations in the study sites. This means that any allele introduced spreads fast among the *P. falciparum* populations with a negative impact on control measures if it is undesirable.

However, this also implies that interbreeding occurs more freely between 2 populations, as studied, which is consistent with the finding of three RH5 haplotypes. Thus, HABP 36727, with no clear evidence of regional variation indicates that most of the malaria infections were actually originating from strains circulating inside Lagos, suggesting limited chances of finding indications of PfRH5 polymorphisms. This has been observed as a potential limitation of this study.

Apart from immunity, occurrence of ancillary causes of selection which may operate on the parasite at other stages of development, is an important limitation of applying genetic technique [21]. These may include rapidly increased frequency of antigenic alleles at a locus which occasionally occurs per chance and may be indistinguishable from selection events. Alleles existing at a locus for a period of time can acquire individual sets of neutral mutations that are unique unless recombination events occur causing linkage between the functionally different alleles.

An important factor to consider in the selection of PfRH5 for involvement in the multi-component malaria vaccine design is the location of epitopes, in conserved regions or/and intrinsically unstructured regions (IURs) of the protein [22, 24]. IURs are important targets of host immune system, including antibody recognition. Disordered epitopes are usually smaller, but more efficient and sensitive to epitope sequence variations [24]. It is therefore imperative to identify population-specific polymorphic immunological hotspots within the disordered antigen–antibody interface from our study population. Although C203Y mutation was most frequent, only one of the codons (197) within the PfRH5–basigin interface was observed to be polymorphic. These findings show a high level of conservation of PfRH5 in comparison with the expectations for a protein with such critical functional role. However, the presence of polymorphisms in the predicted immunologically important peptide requires further attention to understand the impact on the effectiveness of PfRH5-based vaccine.

This study revealed epitopes that are conserved and located in disordered regions on the RH5 sequences from Lagos Nigeria. The inhibition activity of these epitopes or fragments should be assessed by functional assays to ascertain their biological and immunological relevance as candidate epitopes of a PfRH5-based vaccine.

Recognition of a peptide by T cells requires a number of steps, most notably the binding of the peptide to a host-encoded HLA molecule. It was also observed that not significant number of SNPs were found on the epitope regions of PfRH5, which could be inferred to reveal no categorical commitment to populating polymorphisms along these regions. However, larger study populations may be required to confirm this finding.

## Conclusion

Globally efficacious RH5-based vaccine may be considerably protective in Nigeria in spite of the complex genetic diversity of Nigerian isolates of *P. falciparum.* Identification of variants herein reported, is suggestive of the necessity of a wider coverage of studies on the potential efficacy of PfRH5-based vaccine in the study communities in relation to the spatial distribution of immunologically significant genetic mutations in isolates from Nigeria.

## Supplementary information

**Additional file 1: Table S1.** Mutations in PfRH5 sequences from Lagos, Nigeria.

## Data Availability

All data generated or analysed during this study are included in this published article. The nucleotide sequences of *P. falciparum* RH5 gene obtained from this study have been submitted in GenBank database under the Accession Numbers: KX418644–KX418646.

## Abbreviations

RH5: reticulocyte-binding protein homolog 5
gDNA: genomic deoxyribonucleic acid
HABPs: high affinity binding polypeptides
LD: linkage disequilibrium
MHC: major histocompatibility complex
HLA: human leukocyte antigen
IUR: internally unstructured regions
MEGA: Molecular Evolutionary Genetics Analysis
