# Supplementary material for: Genetic polymorphisms in malaria vaccine candidate Plasmodium falciparum reticulocyte-binding protein homologue-5 among populations in Lagos, Nigeria
Source: Malar J. 2020 Jan 6;19:6. doi: 10.1186/s12936-019-3096-0 (PMC6945540; doi:10.1186/s12936-019-3096-0)
Supplement: Supplementary file 1 — Additional file 1: Table S1. Mutations in PfRH5 sequences from Lagos, Nigeria. [file 12936_2019_3096_MOESM1_ESM.docx]

**Haplotype No of Isolates polymorphic Codons Mutated Amino Acids**

**involved**

**H1 45 62,197,240 (K/R), (K/N), (H/R)**

**H2 68 203, 197,81 (C/Y), (K/N),(T/Q)**

**H3 82 62,240,203 (K/R), (H/R)**
